# A comparative study of the complications of surgical tracheostomy in morbidly obese critically ill patients

**DOI:** 10.1186/cc5147

**Published:** 2007-01-12

**Authors:** Ali A El Solh, Wafaa Jaafar

**Affiliations:** 1 Division of Pulmonary, Critical Care, and Sleep Medicine, State University of New York, 462 Grider Street, Buffalo, NY 14215, USA

## Abstract

**Introduction:**

There is little objective comparative information about the postoperative complications of tracheostomy in morbidly obese patients. The aim of this study was to determine the incidence and severity of complications associated with open tracheostomy in critically ill morbidly obese patients during hospitalization.

**Methods:**

During a six year period, all consecutive morbidly obese patients (body mass index [BMI] of greater than or equal to 40 kg/m^2^) who underwent an elective open tracheostomy were compared to a control group (BMI of less than 40 kg/m^2^) of the same institution. Variables examined included age, gender, BMI, Charlson index, and reasons for tracheostomy. All postoperative tracheotomy-related complications that occurred during hospitalization, including death, were recorded.

**Results:**

A tracheostomy was performed in 89 morbidly obese patients out of 427 critically ill patients. A total of 27 complications were recorded in 22 morbidly obese patients (25%) compared to 65 complications in 49 patients (14%) of the control group (*p *= 0.03). The majority of these complications were minor in origin. Overall, nine serious events were responsible for two deaths in the morbidly obese compared to seven cases and two deaths in the control group (*p *= 0.001). Life-threatening complications were attributed to tube obstruction and malpositioning of the tracheostomy after being dislodged. In multivariate analysis, morbid obesity (odds ratio 4.4, 95% confidence interval 2.1 to 11.7) was independently associated with increased risk of tracheostomy-related complications.

**Conclusion:**

In the present series, morbid obesity is associated with increased frequency and life-threatening complications from conventional tracheostomy. Special techniques and operative policies must be applied to overcome loss of airway control.

## Introduction

Tracheostomy continues to be the standard procedure for management of long-term ventilator-dependent patients. It presents several advantages over endotracheal intubation, including lower airway resistance, smaller dead space, less movement of the tube within the trachea, greater patient comfort, and more efficient suction [1,2]. Despite the controversy as to the proper time to perform tracheostomy in critically ill patients, prospective studies suggest that there may be a benefit to early tracheostomy [3]. Yet in the absence of valid evidence based on randomized controlled trials, the decision to place a tracheostomy is made in consideration of the benefits versus the risks of the procedure. Tracheostomy has been associated with serious complications, including tracheal stenosis, increased bacterial colonization, and fatal hemorrhage [4,5]. When it comes to morbidly obese patients, most of the risks and benefits of tracheostomy are not precisely known.

Numerous publications have reported on the safety and complications of percutaneous tracheostomy compared to open tracheostomy in critically ill morbidly obese patients [6,7]. These reports have ranged from increased complications to comparable safety profile. However, to our knowledge, there have been no data addressing the rate of complications of open tracheostomy in morbidly obese compared to non-morbidly obese critically ill patients. The purpose of this study was to document the postoperative complications associated with traditional tracheostomy in our hospitalized population with emphasis on morbid obesity.

## Materials and methods

### Study population

Our study population was derived from an electronic database of all consecutive patients who underwent a conventional open tracheostomy at the Erie County Medical Center (Buffalo, NY, USA) between May 1999 and September 2005. The facility is a tertiary care center affiliated with the State University of New York and serves as a level I trauma and regional burn center for a population of 750,000. The facility includes medical, surgical, cardiac, burn, and open heart units. Exclusion criteria included a history of previous tracheotomy, neck surgery, and cervical irradiation. The study was approved by the local institutional review board, which waived the need for an informed consent.

### Surgical procedure

All tracheotomies were performed according to the technique previously described by Heffner and colleagues [8]. The procedures were performed under general anesthesia in the operating room by a member of the otolaryngology division. Briefly, after dissection of the subcutaneous tissue and underlying muscles, a horizontal incision was made between the second and third tracheal rings. The endotracheal tube was subsequently removed and a cannula was inserted into the distal trachea under visual control. The skin was then sutured on both sides. A chest radiograph was obtained immediately after the completion of the procedure and again the following day.

### Data collection

Data collected were comprised of age, gender, height and weight, Charlson index [9], admission diagnosis, APACHE II (Acute Physiology and Chronic Health Evaluation II) [10] score on intensive care unit (ICU) admission, duration of mechanical ventilation prior to tracheostomy, indication of tracheostomy, early and late complications until hospital discharge, and outcome. Suggested definitions of tracheostomy complications are provided in Table 1. Complications were classified into one of the following categories: early complications, when occurring during the first seven days after the procedure, and late complications, when diagnosed after the seven day period until hospital discharge or death. Each complication was graded as major or minor, according to its clinical relevance. A complication was defined as minor when it caused mild or moderate discomfort. A major complication resulted in severe sequelae or life-threatening lesions. Irrespective of its severity, a complication was considered only once during the follow-up period. Morbid obesity was defined as a body mass index (BMI) of greater than or equal to 40 kg/m^2^.

**Table 1:** Definitions of tracheostomy complications

Minor complications	Definition
Cuff leak	Failure of tracheal cuff to remain inflated at recommended pressure
Minor bleeding	Requiring dressing change, direct pressure, or suture placement
Minor barotrauma	Subcutaneous emphysema
Minor stoma infection	Localized infection treated with topical or systemic antibiotics

Serious complications	

Tube obstruction	Related to clot, mucus, tracheal wall leading respiratory arrest or to severe hypoxemia requiring reintubation
Severe stoma infection	Systemic infection requiring treatment for sepsis or surgical debridements
Major bleeding	Hemoglobin decrease of greater than or equal to 2 g/dl, transfusion of greater than or equal to 2 units of packed red cells, or re-exploration to control bleeding
Major barotrauma	Mediastinal emphysema or pneumothorax
Posterior tracheal wall injury	Injury to membranous trachea from scalpel, or tracheostomy tube
Extratracheal placement	False passage or paratracheal placement of tracheostomy tube
Injury to nerve, artery, or vein	Complications identified and requiring open intervention
Esophageal injury/fistula	Identified and repaired intraoperatively
Tracheal stenosis	Revision and reconstruction
Thyroid injury	Requiring lobe or gland removal

### Statistical analysis

Parametric interval data were analyzed using a two-tailed Student's *t *test. These data are reported as mean ± standard deviation. Nonparametric data were examined using a Mann-Whitney *U *test or Kruskal-Wallis test as appropriate. Nominal data were analyzed by χ^2 ^analysis with Yates continuity correction or Fisher's exact test where appropriate. Demographic, social, and clinical factors found to be significantly different in univariate analysis at a *p *value of less than 0.2 were entered into a stepwise forward logistic regression to assess potential risk factors associated with tracheostomy-related complications. All potential explanatory variables included in the multivariable analyses were subjected to correlation matrix for analysis of collinearity. Statistical significance was defined as a *p *value of less than 0.05. Analyses were performed using SPSS 12.0 software (SPSS Inc., Chicago, IL, USA).

## Results

During the study period, 455 patients underwent tracheostomy during their stay in the ICU. Twenty-eight patients were excluded because of previous history of tracheostomy (*n *= 15), neck surgery (*n *= 9), and cervical irradiation (*n *= 4). Of the 427 tracheostomies, 89 were performed in morbidly obese patients. Table 2 displays the characteristics of the study population. The two cohorts differed in age, BMI, and burden of comorbidity but were similar in gender and severity of illness on admission to the ICU. The most frequent underlying diagnoses for the need of critical care for the study population included pneumonia (21%), obstructive lung diseases (asthma and chronic obstructive pulmonary disease) (14%), postoperative non-vascular surgery (14%), and trauma and burn (13%). Only hypercapnic respiratory failure was reported more frequently in the morbidly obese group than in the control group (*p *< 0.001). Similarly, prolonged mechanical ventilation was more likely to be listed as the indication for tracheostomy in the morbidly obese group and failure to wean was more likely to be listed as the indication for tracheostomy in the control group. Nine tracheostomies were performed on an emergent basis, and two of these were in the morbidly obese group. The duration of mechanical ventilation prior to tracheostomy as well as the number of endotracheal intubations were comparable between the two groups.

**Table 2:** Characteristics of the study population

	Morbidly obese (*n *= 89)	Control (*n *= 338)	*P *value
Age (years)	51.1 ± 9.4	57.9 ± 18.1	0.02
Gender (male/female)	52/37	198/140	0.9
Body mass index (kg/m^2^)	48.6 ± 8.1	29.3 ± 5.3	< 0.001
Charlson index	2.1 ± 1.1	0.9 ± 1.0	< 0.001
APACHE II score	18 ± 4	19 ± 6	0.5
Reasons for ICU admission			
Obstructive lung disease	7 (8%)	51 (15%)	0.11
Pneumonia	17 (19%)	72 (21%)	0.76
Congestive heart failure	6 (7%)	42 (12%)	0.19
Hypercapnic respiratory failure	15 (17%)	7 (2%)	< 0.001
Sepsis	13 (15%)	33 (10%)	0.26
Cerebrovascular accident	3 (4%)	17 (5%)	0.71
Cardiothoracic/vascular surgery	6 (7%)	14 (4%)	0.45
Non-vascular surgery	12 (13%)	47 (14%)	0.94
Trauma/burn	8 (9%)	49 (14%)	0.24
Others	2 (2%)	5 (1%)	0.97
Indications for tracheostomy			
Prolonged mechanical ventilation	46 (52%)	105 (31%)	< 0.001
Failure to wean	34 (38%)	192 (57%)	0.003
Vocal cord paralysis	0	7 (2%)	0.37
Facial trauma	9 (10%)	24 (7%)	0.47
Epiglottitis	0 (1%)	10 (3%)	0.21
Duration of mechanical ventilation prior to tracheotomy (days)	11.7 ± 5.2	12.8 ± 6.9	0.35
Endotracheal intubations prior to tracheostomy (days)	2 ± 1	2 ± 1	0.78

APACHE II, Acute Physiology and Chronic Health Evaluation II; ICU, intensive care unit.

A total of 27 complications were recorded in 22 patients (25%) of the morbidly obese group compared to 65 complications in 49 patients (14%) of the control group (*p *= 0.03). Five morbidly obese patients had two complications, whereas 15 controls had two complications and one control had three. The severity and time period of complications for both study groups are detailed in Table 3.

**Table 3:** Early and late complications of tracheostomy

	Morbid obesity (*n *= 89)		Control (*n *= 338)	
Complication	Early	Late	Early	Late
Cuff leak	1 (1%)	2 (2%)	6 (2%)	18 (5%)
Tube obstruction	1 (1%)	3 (3%)	0	1 (0.3%)
Minor bleeding	9 (10%)	1 (1%)	22 (7%)	1 (0.3%)
Major bleeding	0%	1 (1%)	1 (0.3%)	5 (2%)
Minor barotrauma	1 (1%)	0	1 (0.3%)	0
Major barotrauma	0	0	1 (0.3%)	0
Minor stoma infection	3 (4%)	1 (1%)	7 (2%)	2 (0.5%)
Severe stoma infection	0	1 (1%)	0	0
Tracheoesophageal fistula	0	0	0	0
Extratracheal placement	1 (1%)	2 (2%)	0	0
Tracheal stenosis	0	0	0	0
Injury to nerve, artery, or vein	0	0	0	0

Minor bleeding was the most frequently reported complication in both groups (11% versus 7%; *p *= 0.24). Ninety-four percent of the cases (31 out of 33 cases) occurred during the first seven days postoperatively. In all of these instances, bleeding was controlled with light packing. Cuff leak represented the second most common complication in the study population (3% in the morbidly obese and 7% in the control group; *p *= 0.26), but unlike minor bleeding, these events were noted primarily after seven days of tracheostomy placement. Whereas cuff malfunctioning was responsible for early failure, loss of tracheal wall rigidity secondary to prolonged mechanical ventilation was responsible for the late complication in both cohorts.

Morbidly obese patients were particularly at higher risk for serious life-threatening complications. Overall, nine serious events were responsible for two deaths compared to seven cases and two deaths in the control group (*p *= 0.001 for serious events). Tube obstruction was the culprit in four of the nine morbidly obese cases. An early case was attributed to a blood clot after the patient had evidence of minor bleeding. The patient developed severe hypoxemia but the event was detected early while the patient was still in the ICU. In contrast, the other three cases occurred outside the critical care unit between 7 and 18 days after liberation from mechanical ventilation. Despite delivery of high humidity, two patients were found to have a mucous plug that led to severe hypoxemia and severe bradycardia. Anoxic encephalopathy ensued in both patients; in one case, the family requested termination of life support, whereas the other patient required transfer to a long-term care facility. Of interest, all three cases had non-fenestrated cuffed synthetic tubes in place. In the control group, one patient with reduced consciousness secondary to head trauma sustained a respiratory arrest after a mucous plug and did not survive resuscitation.

Accidental decannulation followed by extratracheal tube placement (false lumen) was the next most serious complication reported in the critically ill morbidly obese patients. Whereas none of the control group was identified with this complication, three morbidly obese patients had serious consequences from attempting to reinstate the tracheostomy tube. One complication occurred five days postoperatively after the patient removed the tube while on mechanical ventilation. The patient developed massive subcutaneous emphysema that resulted in bilateral tension pneumothorax and cardiorespiratory arrest. The other two complications developed 11 and 28 days after surgery when attempting to replace or downsize the tracheostomy tube. In both instances, orotracheal intubation was performed after both patients went into respiratory distress. A revision of the tracheostomy was performed subsequently without further complications.

The incidence of major bleeding was not significantly different between the two groups. One morbidly obese and four control patients had a decrease of hematocrit of more than 2 g/dl in the first 48 hours postoperatively, which was attributed to extensive oozing around the site of the wound. Bedside hemostasis was achieved by local packing and application of thrombin. Two control patients who had significant bleeding at 16 and 38 days after surgery were suspected of developing a tracheoinnominate artery fistula. One patient had a massive aspiration and could not be resuscitated. The other patient was transferred to the operating room, where an immediate exploration was performed and ligation of the bleeding vessel was conducted.

The rate and timing of stoma infection were also comparable between the two groups. Thirteen patients had local wound infection that was reported between days 2 and 10 of mechanical ventilation. Cultures of the wound showed predominance of gram-negative bacilli, notably *Serratia marcescens *(*n *= 1), *Escherichia coli *(*n *= 5), and *Pseudomonas aeruginosa *(*n *= 6). Apart from local antibiotic application, none of these patients required systemic antimicrobial therapy to treat the infection. Only one morbidly obese patient was found to have a paratracheal abscess after persistent fever that was unresponsive to systemic antimicrobial therapy. A computer tomography was diagnostic of the abscess, and the patient required prompt drainage followed by four weeks of therapy targeted toward gram-negative and anaerobic pathogens. None of our study population had tracheoesophageal fistula or injury to a nerve, artery, or vein during the postoperative period. Finally, no incidence of tracheal stenosis was observed during the length of hospitalization in either group.

Three factors (age, BMI, and Charlson index) found to be significant in univariate analysis were entered into multivariate analysis. Only BMI (odds ratio 4.4, 95% confidence interval 2.1 to 11.7) was independently associated with increased risk of tracheostomy-related complications.

## Discussion

The peri- and postoperative complications associated with surgical tracheostomy have been dramatically reduced since this technique was described initially. In the obese patient, however, special anatomic considerations make this procedure a challenging operation. In the current study, the incidence of complications related to tracheostomy in the morbidly obese was 25% with an estimated mortality of 2%. The majority of these complications were minor in origin; however, life-threatening complications were more common than in the comparator group and were attributed mainly to the loss of airway accessibility.

Obstruction of the tracheotomy tube is a commonly reported event in the postoperative period. When it occurs in the first 24 hours, it is usually the result of tube impingement on the posterior tracheal wall, partial displacement into the mediastinum, a blood clot, or mucous plug. For the morbidly obese patient who is lying supine and partially sedated, hypoxemia develops rapidly as a consequence of reduced expiratory reserve volume [11]. To avert anoxic encephalopathy, immediate resuscitation is required. As a result, all morbidly obese patients in our health care institution are monitored in an ICU setting for at least 72 hours even when mechanical ventilation has been discontinued. Nonetheless, the risk of developing this complication persists beyond this time frame and the catastrophic sequelae of two out of the three morbidly obese patients in our study underline the need for close monitoring. Despite the deflation of the tracheostomy cuff in all three cases prior to their transfer to the ward, the obstruction of the tracheostomy tube is thought to be partially the result of the distorted anatomical neck structure of the morbidly obese, which may limit adequate air entry. It is plausible that the relative narrowing of the cervical tracheal area compared to the non-obese [12] maintains a tight seal when a deflated cuffed Shiley tube remains in place. Adding to the complexity of the situation, submental fat deposition that may reach below the sternal notch could occlude the outer opening of the standard tracheostomy, rendering any oxygenation extremely limited or nonexistent. Simmons [13] recommended the application of an elastic bandage or a Barton bandage to move the chin out of the way. Others have considered the use of an extension attached to the outer opening [14]. We have instituted a policy of replacing the cuffed Shiley tube with a metal tracheostomy tube on all morbidly obese patients once the tracheostomy tract is well formed. Since the implementation of such policy, we have not recorded any catastrophic obstructive event.

The insertion of a loosely attached tracheal tube, however, can lead to decannulation and reinsertion complications. The gravity of decannulation in morbidly obese patients is emphasized by the fact that this event is associated with 30% mortality in this series. Morbidly obese patients with short, thick necks usually have too much soft tissue between the trachea and the skin. Unsuccessful blinded reintubation attempts may cause tube misplacement in the pretracheal fascia with resultant tracheal compression and respiratory arrest. Some surgeons advocate performing a Björk flap at the time of surgery [15] to prevent this complication. The procedure involves incising an inverted U-shaped flap in the anterior tracheal wall at the second to fourth cartilaginous rings. The flap is reflected downward and outward with the upper border sutured to the skin, creating a bridge of tracheal tissue that guides tube replacement and avoids creation of a false channel [15,16]. Opponents of this technique have argued that tracheal flaps were associated with higher incidence of tracheal stenosis after decannulation [17], but long-term follow-up failed to substantiate this argument [18]. Alternatively, Gross and colleagues [19] advocated a cervical lipectomy in combination with tracheostomy. As to whether morbidly obese patients will benefit from the application of these techniques in reducing the rate of extratracheal placement, there are to our knowledge no studies that provide a conclusive answer. Until a consensus is reached, we have adopted the use of an uncuffed endotracheal tube of a size that would be able to fit through the internal diameter of the tracheostomy tube. The beveled tip of the endotracheal tube assists in proper placement and in providing temporary access for ventilation. If tube placement is not successful, a pediatric laryngoscope is used to allow examination of the wound. If obstruction is ruled out, the tracheostomy tube is then advanced over this obturator airway.

Our study has a number of limitations. First, assurance of accurate documentation of all complications is limited by the retrospective nature of the study. We relied on the accuracy of operative notes and thoroughness of chart documentation. Second, the complications spanned the period of hospitalization only and did not extend beyond hospital discharge. Therefore, we could not adequately assess the rate of tracheal stenosis or tracheomalacia in these patients. Third, the complication rates are derived from a single tertiary care center and may not be applicable to other referral centers. However, our series included the largest number of morbidly obese patients who underwent tracheostomy published so far.

## Conclusion

In summary, the risk of perioperative complications of tracheostomy in the critically ill morbidly obese is higher than in non-morbidly obese patients and can be associated with significant morbidity and mortality. Life-threatening complications are attributed in the majority of cases to loss of airway patency. To avoid catastrophic sequelae, special techniques and operative policies must be applied.

## Key messages

• Conventional tracheostomy in morbidly obese critically ill patients is associated with increased frequency of complications compared to non-morbidly obese patients.

• The majority of complications are minor in origin.

• Life-threatening complications are attributed to dislodging and obstruction of the tracheostomy tube.

• Special techniques and operative policies must be instituted to avoid catastrophic complications.

## Abbreviations

BMI = body mass index; ICU = intensive care unit.

## Competing interests

The authors declare that they have no competing interests.

## Authors' contributions

AES conceived of the study, reviewed all statistical analysis, and drafted and edited the manuscript. WJ conducted the literature search, collected the data, and performed the statistical analysis. Both authors read and approved the final manuscript.
